# Network meta-analysis and cost-effectiveness analysis comparing Cetuximab-β and Cetuximab for Chinese patients with RAS/BRAF wild-type metastatic colorectal cancer

**DOI:** 10.3389/fphar.2025.1568385

**Published:** 2025-09-15

**Authors:** Rongjun Tong, Mengyu Yang, Wanjie Zhang, Mengyuan Zhou, Linning Wang, Yun Lu, Feng Chang

**Affiliations:** Center for Healthcare Policy Research, School of International Pharmaceutical Business, China Pharmaceutical University, Nanjing, China

**Keywords:** Cetuximab-β, Cetuximab, metastatic colorectal cancer, network meta-analysis, cost-effectiveness analysis

## Abstract

**Background:**

The escalating economic burden of metastatic colorectal cancer (mCRC) in China necessitates cost-effective first-line treatments. Cetuximab-β, a newer version of Cetuximab, is approved for first-line RAS/BRAF wild-type mCRC. This study evaluates the cost-effectiveness of Cetuximab-β with FOLFIRI for mCRC patients, comparing it to Cetuximab plus chemotherapy to guide clinical decision-making and policy development.

**Methods:**

We conducted a network meta-analysis (NMA) of six randomized controlled trials to compare overall survival (OS), progression-free survival (PFS), and adverse events (AEs). Subsequently, a cost-effectiveness analysis (CEA) was performed using a 10-year partitioned survival model from the Chinese healthcare payer perspective. Costs were standardized to 2024 US dollars ($1 = ¥7.25). Both costs and outcomes were discounted annually at 5%. The model estimated life-years (LYs), quality-adjusted life-years (QALYs), total costs, and incremental cost-effectiveness ratios (ICER). Model uncertainty was evaluated via one-way sensitivity analysis, probabilistic sensitivity analysis, and scenario analyses.

**Results:**

The NMA showed comparable efficacy between Cetuximab-β and Cetuximab, with Cetuximab demonstrating an OS HR of 1.10 (95% CI 0.67–1.90) and a PFS HR of 0.94 (95% CI 0.49–1.80) compared with Cetuximab-β, along with a trend towards a more favorable safety profile for Cetuximab-β. CEA showed Cetuximab-β reduced costs by $12,005.54 ($34,996.43 vs. $47,001.97) and gained 0.10 QALYs (1.90 vs. 1.80 QALYs) *versus* Cetuximab, yielding a dominant ICER (-$120,743/QALY). Sensitivity and scenario analyses confirmed robustness.

**Conclusion:**

Cetuximab-β plus FOLFIRI represents a dominant, cost-saving strategy compared to Cetuximab plus chemotherapy for first-line treatment of RAS/BRAF wild-type mCRC in China.

## Introduction

Colorectal cancer (CRC) is a pressing global health issue due to its high incidence and mortality rates. Globally, CRC ranks as the third most commonly diagnosed cancer and the second leading cause of cancer-related deaths, with an estimated 1.93 million new cases and 903,900 deaths annually (Bray et al., 2024). In China, CRC poses a substantial burden, ranking second in incidence and fourth in mortality, accounting for approximately 517,100 new cases and 240,000 deaths each year (Han et al., 2024). These statistics highlight the urgent need for optimized treatment strategies and targeted resource allocation to mitigate the CRC burden worldwide and in China.

The nonspecific symptoms of CRC, such as diarrhea, constipation, rectal bleeding, and abdominal pain, often lead to delayed diagnoses (Holtedahl et al., 2021). As a result, many patients are diagnosed at advanced stages, frequently accompanied by distant metastases. Approximately 20% of CRC cases are metastatic at diagnosis, and an additional 25% of initially localized cases eventually progress to metastatic colorectal cancer (mCRC) (Biller and Schrag, 2021; Kelly et al., 2014). At this stage, curative surgical options are typically unavailable, and prognosis remains poor.

The RAS gene is a key determinant in selecting first-line treatment for mCRC, with about 50% of mCRC patients exhibiting RAS gene mutations (NCCN, 2024). Despite advancements in treatment, including the development of targeted therapies and immunotherapies like PD-1 inhibitors, the overall survival for patients remains low, particularly for those with BRAF mutations or mismatch repair-deficient cancers (Yang et al., 2023). For patients with RAS wild-type (WT) mCRC, early clinical studies have demonstrated the efficacy of Cetuximab, a recombinant anti-epidermal growth factor receptor (EGFR) monoclonal antibody, when combined with FOLFIRI (a chemotherapy regimen combining calcium folinate, fluorouracil, and irinotecan) or FOLFOX (a chemotherapy regimen including calcium folinate, fluorouracil, and oxaliplatin). Cetuximab-β, a newer version of Cetuximab, is produced in Chinese hamster ovary (CHO) cells rather than the mouse SP2/0 cell line used for Cetuximab. Unlike the SP2/0 cell line, CHO cells lack the gene for α-1,3-galactosyltransferase, resulting in a glycosylation profile for Cetuximab-β that contains lower levels of galactose-α-1,3-galactose (Gal (α 1-3) Gal) (Chung et al., 2008). Since pre-existing IgE antibodies targeting Gal (α 1-3) Gal have been associated with adverse reactions (AEs) to Cetuximab, Cetuximab-β may exhibit a reduced incidence of AEs (Chinese Society of Clinical Oncology Colorectal Cancer Expert Committee, 2024; Wang and Guo, 2017; Lammerts van Bueren et al., 2011).

Recently, Cetuximab-β was approved for use in combination with FOLFIRI as a first-line treatment for patients with mCRC based on the results of a phase III clinical trial (009mCRCIIIP) (FOLFIRI, 2024). However, its cost-effectiveness compared to existing treatments has not yet been fully assessed. This study aims to conduct a pharmacoeconomic evaluation of Cetuximab-β plus FOLFIRI for patients with RAS/BRAF wild-type mCRC in China, comparing it with Cetuximab plus chemotherapy. The findings are intended to inform clinical decision-making and guide policy development regarding adopting Cetuximab-β in routine practice.

## Methods

### Network meta-analysis

#### Study selection and assessment of risk of bias

We conducted a systematic literature search in PubMed, the Cochrane Library, and the Web of Science for eligible publications up to 30 June 2024. The search terms were: (Cetuximab-β OR Cetuximab OR FOLFIRI OR FOLFOX) AND (“metastatic colorectal cancer”) AND (“RAS/BRAF wild type”) AND random*. Additionally, we planned to contact the marketing department of the relevant pharmaceutical company to request supplementary data, such as unpublished clinical trial outcomes. The risk of bias for included clinical trials was assessed using RevMan software (version 5.4) following the methodology outlined in the Cochrane Handbook for Systematic Reviews of Interventions.

#### Statistical analysis

The primary outcomes of the network meta-analysis (NMA) include efficacy (Hazard ratios (HR) for overall survival (OS) and progression-free survival (PFS) and safety (relative risk (RR) for grade 3/4 AEs) comparisons between cetuximab β plus FOLFIRI, and Cetuximab plus chemotherapy. HR and their 95% confidence intervals (CIs) for OS and PFS were calculated using R software (version 4.1.1) and the ‘netmeta’ package for each treatment regimen. Due to observed heterogeneity across clinical trials, we applied a random effects model to estimate treatment effects. An indirect comparison of the safety profiles was conducted by calculating the RR and its 95% CI for grade 3/4 AEs, considered severe according to standard clinical trial grading criteria.

### Cost-effectiveness analysis

In this cost-effectiveness analysis, we compared Cetuximab-β plus FOLFIRI with Cetuximab plus chemotherapy, all of which are approved for the treatment of patients with RAS/BRAF WT mCRC in China. A 5% annual discount rate was applied to both costs and effectiveness to account for the time value of money, in line with standard pharmacoeconomic evaluation practices. The primary outcomes measured were life-years (LYs), quality-adjusted life-years (QALYs), overall costs, and incremental cost-effectiveness ratios (ICERs) between the treatment regimens. The willingness-to-pay (WTP) threshold was determined following the China Guidelines for Pharmacoeconomic Evaluations (2020) (Liu et al., 2020), set at three times the *per capita* 2023 Chinese GDP, or approximately $37,023 per QALY. This threshold is used to assess whether a treatment provides sufficient value relative to its cost. The study was conducted in adherence to the Consolidated Health Economic Evaluation Reporting Standards (CHEERS) guidelines (Husereau et al., 2022). As no individual patient-level data were used in the analysis, this study does not qualify as human subjects research and thus did not require review or approval by an institutional review board or ethics committee.

#### Patients and treatments

The study population consisted of patients with RAS/BRAF WT mCRC receiving first-line treatment. Cetuximab-β was administered at an initial dose of 400 mg/m^2^ during the first week, followed by 250 mg/m^2^ weekly thereafter, with Cetuximab given at the same dosage regimen. According to the package insert, Cetuximab-β is approved for use in combination with FOLFIRI, while Cetuximab is approved for use with either FOLFIRI or FOLFOX. The proportion of patients receiving FOLFIRI or FOLFOX was based on data from the study (Xu et al., 2020). In the event of disease progression, patients were switched to subsequent treatments, as detailed in Supplementary Table S1, and received the best supportive care until death. Grade 3/4 AEs, defined as severe or life-threatening, included neutropenia, febrile neutropenia, anemia, hypokalemia, diarrhea, and rash. For this analysis, we assumed a patient weight of 60 kg and a body surface area of 1.6 m^2^ (Huang et al., 2017).

#### Model structure

Partitioned survival modeling (PSM) is particularly suited for oncology applications as it directly utilizes the two most frequently reported trial endpoints - PFS and OS (Llovet et al., 2008; Woods et al., 2020). This approach was preferred to state-transition modeling given its ability to incorporate published Kaplan-Meier data from comparator trials when individual patient-level data are unavailable (Glover et al., 2021). Therefore, A PSM with a 1-month cycle was developed using Excel 2020 in this stady. The PSM included three mutually exclusive health states: PFS, progressive disease (PD), and death (Supplementary Figure 1). The time horizon for the model was set to 10 years, as more than 99% of the cohort was expected to have died within this period.

#### Effectiveness

The survival curves for OS and PFS in patients treated with Cetuximab-β plus FOLFIRI were derived by fitting individual patient data from the 009mCRCIII-P clinical trial (Holtedahl et al., 2021) to a parametric survival model in R software (version 4.1.1) (Zhu et al., 2022), incorporating natural mortality data from the Chinese population to extrapolate long-term outcomes. The Weibull distribution was identified as the best-fitting model based on Akaike’s information criterion (AIC) and Bayesian information criterion (BIC), outperforming alternative models such as log-logistic, Gompertz, exponential, and log-normal distributions (Supplementary Tables S2, S3; Supplementary Figures 2–5) (Latimer, 2013). The shape (γ) and scale (λ) parameters of the Weibull distribution were estimated using R. Survival curves for patients receiving Cetuximab plus chemotherapy were derived by adjusting the survival data of Cetuximab-β plus FOLFIRI using results from the network meta-analysis.

Health utility values for the PFS state and the PD state were assumed to be 0.85 and 0.68, respectively, based on previously published data (Wu et al., 2017). Additionally, the disutility values associated with grade 3/4 AEs were incorporated into the analysis.

#### Cost inputs

In this analysis, direct medical costs were considered from the perspective of Chinese payers. These costs included drug costs, administration costs, management of grade 3/4 AEs with an incidence rate greater than 5%, terminal care, follow-up, and monitoring. The prices for drugs, administration, and part of the follow-up (details of the follow-up program are presented in Supplementary Table S5) and monitoring costs were obtained from the medical and health information service platform of Menet and the official website of the provincial healthcare pricing bureaus of China. The remaining costs were sourced from previously published literature (Table 1). All costs in the study were converted to USD 2,024 based on the exchange rate ($1 = ¥7.25) and consumer price index.

**TABLE 1:** Basic parameters input to the model.

Parameter	Baseline value	Lower limit	Upper limit	Distribution	Source
Weibull survival model of Cetuximab-β group					
OS	shape = 1.324	NA	NA	NA	Model fitting
	scale = 0.008				
PFS	shape = 1.788	NA	NA	NA	Model fitting
	scale = 0.008				
HRs of other regimens *versus* Cetuximab-β group	See the results of the network meta-analysis			NA	NA
Risk of Grade 3/4 AEs					
Cetuximab-β group					FOLFIRI (2024)
Neutropenia	0.57	0.51	0.62	Beta	FOLFIRI (2024)
Febrile Neutropenia	0.05	0.05	0.06	Beta	FOLFIRI (2024)
Hypokalemia	0.04	0.04	0.05	Beta	FOLFIRI (2024)
Anemia	0.05	0.05	0.06	Beta	FOLFIRI (2024)
Diarrhea	0.10	0.09	0.11	Beta	FOLFIRI (2024)
Rash	0.06	0.06	0.07	Beta	FOLFIRI (2024)
RRs of Cetuximab *versus* Cetuximab-β	See the results of the network meta-analysis			Beta	Model fitting
Health utilities					
PFS	0.85	0.77	0.94	Beta	Glover et al. (2021)
PD	0.68	0.61	0.75	Beta	Glover et al. (2021)
Disutility of grade 3/4 AEs utility					
Neutropenia	−0.09	−0.10	−0.08	Beta	Nafees et al. (2008)
Febrile Neutropenia	−0.09	−0.10	−0.08	Beta	Zhu et al. (2022)
Hypokalemia	−0.08	−0.09	−0.07	Beta	Stintzing et al. (2020)
Anemia	−0.07	−0.08	−0.07	Beta	Westwood et al. (2014)
Diarrhea	−0.09	−0.10	−0.08	Beta	Latimer (2013)
Rash	−0.03	−0.04	−0.03	Beta	Zhu et al. (2022)
Cost data, $					
Drug cost					
Cetuximab-β per 100 mg	107.67	96.87	118.40	Gamma	National Healthcare Security Administration (2024)
Cetuximab per 100 mg	148.07	133.26	146.59	Gamma	Menet - Medical and Health Information Service Platform (2024)
Bevacizumab per 100 mg	158.21	142.39	156.62	Gamma	Stintzing et al. (2020)
Irinotecan per 40 mg	7.73	6.96	7.65	Gamma	Stintzing et al. (2020)
Oxaliplatin per 50 mg	42.50	38.25	42.08	Gamma	Stintzing et al. (2020)
Fluorouracil per 250 mg	5.64	5.08	5.58	Gamma	Stintzing et al. (2020)
Calcium Folinate per 300 mg	23.40	21.06	23.16	Gamma	Stintzing et al. (2020)
Cost of Grade 3/4 AEs					
Neutropenia	102.15	91.94	101.13	Gamma	Tang et al. (2023)
Febrile Neutropenia	116.69	105.02	115.53	Gamma	Chen (2023)
Hypokalemia	13.80	12.42	13.66	Gamma	Dong (2023)
Anemia	478.56	430.70	473.77	Gamma	Sun and Li (2022)
Diarrhea	28.46	25.62	28.18	Gamma	Liu and Chen (2021)
Rash	15.88	14.29	15.72	Gamma	Zhang et al. (2015)
Cost of drug administration per cycle	45.35	40.82	44.90	Gamma	See the Supplementary Table S4
Cost of follow-up and monitoring per cycle					
PFS	112.62	101.36	111.49	Gamma	See the Supplementary Table S5
PD	83.90	75.51	83.06	Gamma	See the Supplementary Table S5
Cost of PD per cycle	827.65	744.89	819.38	Gamma	See the Supplementary Table S1
Terminal care per patient	1571.84	1414.66	1556.12	Gamma	Wu et al. (2014)
Discount rate	5.00%	0.00%	8.00%	Uniform	Liu et al. (2020)
Body weight (kg)	60.00	NA	NA	NA	NA
Body surface area (m^2^)	1.60	NA	NA	NA	NA

Abbreviations: OS, overall survival; PFS, Progression-Free Survival; HR, hazard ratio; AE, adverse event; RR, relative risk; PD, progressive disease.

#### Sensitivity analyses

We conducted both one-way sensitivity analysis and probabilistic sensitivity analysis (PSA) to assess the uncertainty of the model results. In the one-way sensitivity analysis, we varied each parameter within its 95% CI reported in the literature. If no CIs were available, we applied a default variance of ±10% from the baseline values. Additionally, PSA was performed using 1,000 Monte Carlo simulations to evaluate the probability of the treatment regimens being cost-effective.

#### Scenario analyses

To evaluate the robustness of the model results, scenario analyses were conducted to test key assumptions and explore the impact of alternative settings. One key assumption was that Cetuximab-β, developed as an improved formulation of Cetuximab, is equivalent to Cetuximab in terms of efficacy and safety. A scenario analysis was performed to assess the cost-effectiveness outcomes under this assumption of equivalence. Additionally, the impact of varying the model’s time horizon was examined by testing time horizons of 5, 15, 25, and 35 years to assess the long-term cost-effectiveness of the treatment regimens.

## Results

### Network meta-analysis

The NMA included data from six RCTs, (FOLFIRI, 2024; Qin et al., 2018; Bokemeyer et al., 2009; Tveit et al., 2012; Bokemeyer et al., 2011; Van Cutsem et al., 2015) comprising three phase II and three phase III studies, with a total of 1,750 patients (Supplementary Table S6). A comparison of baseline characteristics across the studies is provided in Supplementary Table S7, the literature screening procedure is shown in Supplementary Figure S6, and the NMA model diagram is shown in Supplementary Figure S7. Of the study population, 257 patients received Cetuximab-β, 579 received Cetuximab, and 869 received chemotherapy alone. The risk of bias assessment is shown in Supplementary Figure S8.

The NMA indicated that mCRC patients receiving Cetuximab-β plus FOLFIRI had reduced PFS (HR 0.94; 95% CI 0.49–1.80 for Cetuximab vs. Cetuximab-β) compared to those treated with Cetuximab plus chemotherapy, but nevertheless demonstrated longer OS (HR 1.10; 95% CI 0.67–1.90 for Cetuximab vs. Cetuximab-β), though neither outcome reached statistical significance. The incidence of diarrhea was significantly lower in the Cetuximab-β group than in the Cetuximab group (RR = 1.90, 95% CI, 1.04–3.47). No significant differences were observed between the two groups for the other five AEs (Table 2).

**TABLE 2:** Result of Network meta-analysis (compared to Cetuximab-β).

Treatments	Cetuximab	Chemotherapy
HR (95% CI)		
OS	1.1 (0.67, 1.9)	1.4 (0.86, 2.2)
PFS	0.94 (0.49, 1.8)	1.6 (0.89, 2.8)
RR (95% CI)		
Neutropenia	0.71 (0.44, 1.16)	0.61 (0.40, 0.94)
Febrile Neutropenia	0.81 (0.24, 2.69)	0.56 (0.23, 1.38)
Hypokalemia	0.52 (0.20, 1.36)	1.33 (0.38, 4.64)
Anemia	2.23 (0.65, 7.68)	1.43 (0.72, 2.87)
Diarrhea	1.90 (1.04, 3.47)	1.24 (0.76, 2.02)
Rash	1.79 (0.26, 12.11)	0.09 (0.02, 0.50)

Abbreviations: OS, overall survival; PFS, Progression-Free Survival; HR, hazard ratio; RR, relative risk.

### Cost-effectiveness analysis

#### Base-case analysis

Over a 10-year time horizon, treatment with Cetuximab-β resulted in a cost of $34996.43, providing 1.90 QALYs and 2.73 LYs. In contrast, Cetuximab incurred a cost of $47001.97, providing 1.80 QALYs and 2.54 LYs (Table 3). The ICER for Cetuximab-β compared to Cetuximab was negative (-$120742.96 per QALY), reflecting cost savings with Cetuximab-β.

**TABLE 3:** Summary of base-case analyses.

Treatments	Cetuximab-β	Cetuximab
QALYs during PFS	0.94	0.97
QALYs during PD	0.96	0.83
QALYs of Grade 3/4 AEs	−0.01	−0.01
LYs	2.73	2.54
Total QALYs	1.90	1.80
Cost of medication, $	31108.50	43080.35
Cost of Grade 3/4 AEs management, $	92.84	108.47
Cost during PD, $	611.61	570.38
Cost of follow-up, $	1621.87	1677.12
Cost of terminal care, $	1561.62	1565.66
Total cost, $	34996.43	47001.97
ICER	−120742.96	

Abbreviations: QALY, Quality-Adjusted Life Year; PFS, Progression-Free Survival; PD, progressive disease; AE, adverse event; ICER, Incremental Cost-Effectiveness Ratio.

#### Sensitivity analysis

**FIGURE 1:**
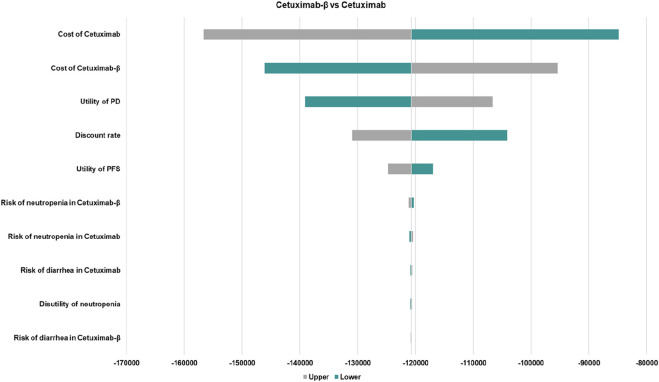
The one-way sensitivity analyses of Cetuximab-β vs. Cetuximab. PFS, Progression-Free Survival; PD, Disease Progressed. Only the top ten parameters with the greatest impact on the results are shown.

In comparing Cetuximab-β with Cetuximab, the results of the one-way sensitivity analysis revealed that parameters such as the cost of Cetuximab and Cetuximab-β, and the utility value for the PD and PFS had the greatest impact on the base case results (Figure 1). Nevertheless, the ICER values obtained after varying all parameters remained below the WTP threshold, indicating that these changes did not alter the conclusions of the analysis, thus confirming the robustness of the base case results.

**FIGURE 2:**
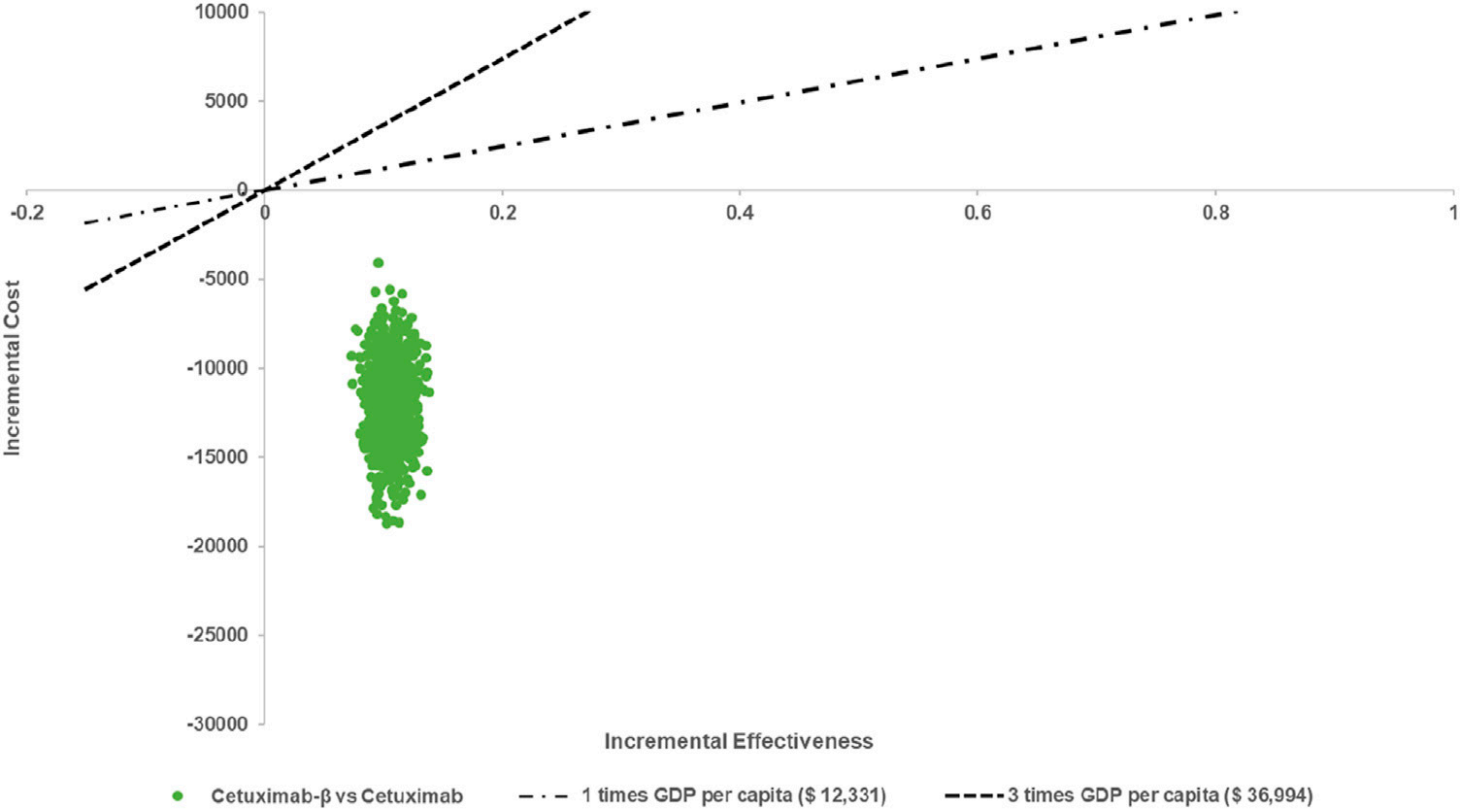
The results of the Monte Carlo probabilistic sensitivity analysis.

**FIGURE 3:**
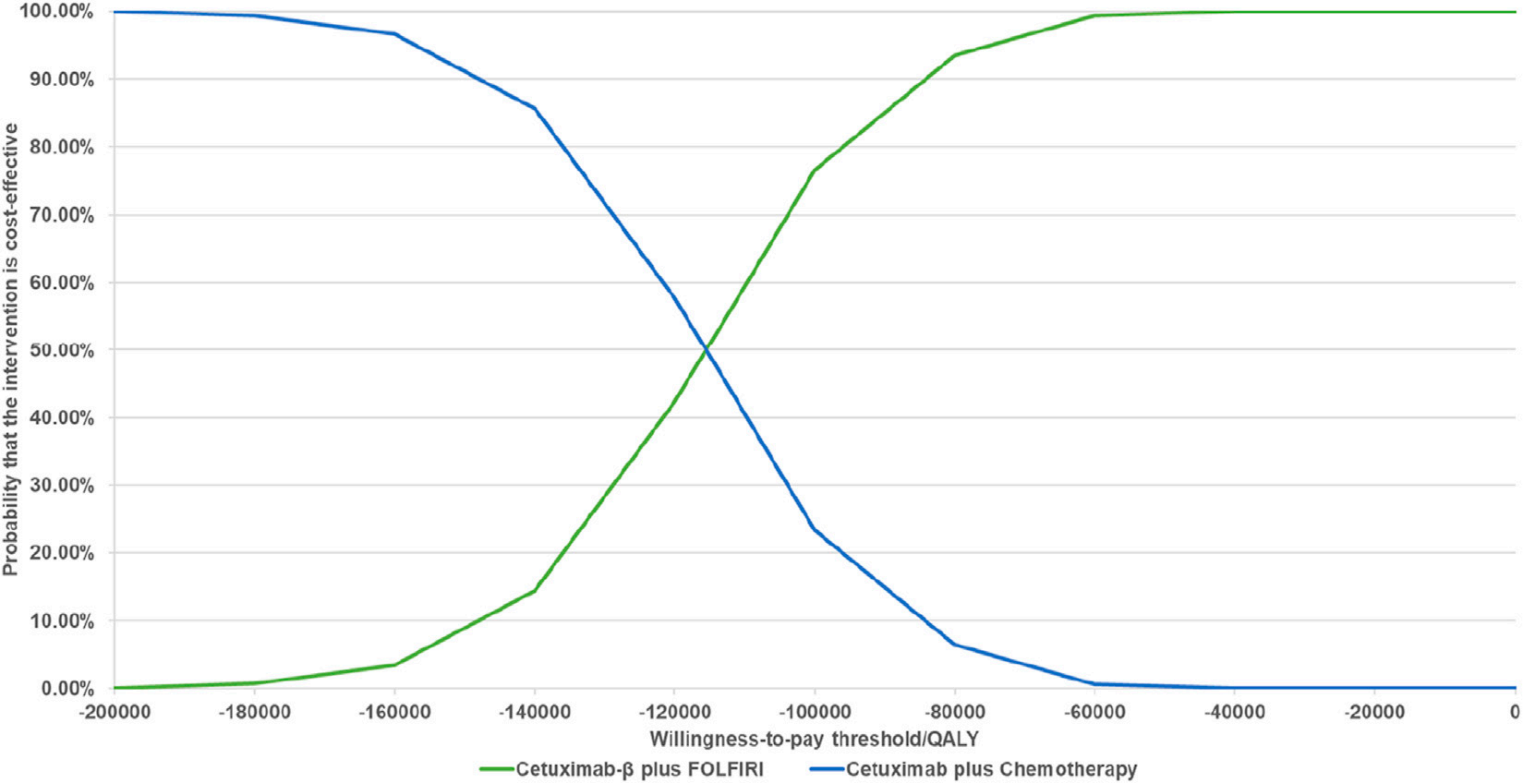
Cost-effectiveness Acceptability Curves (When the WTP is less than 3 times *per capita*, Cetuximab β is 100% cost-effective compared with Cetuximab).

The results of the PSA indicated that, when comparing Cetuximab-β to Cetuximab, Cetuximab-β had an 100% probability of being cost-effective under WTP threshold (Figures 2, 3).

#### Scenario analyses

The scenario analysis results indicated that, under the assumption of equivalence between Cetuximab-β and Cetuximab, the incremental cost of Cetuximab-β compared with Cetuximab was -$10,567.38, suggesting a cost saving (Table 4). This cost difference was primarily attributed to the lower price of Cetuximab-β, as well as its specific administration with FOLFIRI, which is less expensive compared to FOLFOX. In contrast, Cetuximab can be administered with either FOLFIRI or FOLFOX, contributing to the higher overall cost for Cetuximab.

**TABLE 4:** The scenario analysis results under the assumption of equivalence between Cetuximab-β and Cetuximab.

Treatments	Cetuximab-β	Cetuximab
Total Cost, $	34,996.43	45,563.81
Cost of Monoclonal antibody, $	25,123.81	34,561.34
Cost of Chemotherapy, $	5,264.35	6,461.42
Cost of grade 3/4 AEs, $	92.84	92.84
Cost during PD, $	611.61	611.61
ΔC, $	−10,567.38	

Abbreviations: AE, adverse event; PD, progressive disease.

The scenario analysis evaluating different study time horizons demonstrated that extending the time horizon from 10 to 35 years did not significantly alter the outcomes compared to the base case analysis (Supplementary Figure S9). These findings support the appropriateness of using a 10-year time horizon for the primary analysis.

## Discussion

In recent years, the economic burden of CRC has increased, with healthcare spending per patient rising significantly (Wang et al., 2023). Studies indicate that direct healthcare expenditure for CRC patients in China now exceeds the country’s GDP *per capita* in the same year (Huang et al., 2017). This growing economic burden is driven in part by the high mortality associated with mCRC, which remains one of the leading causes of death among CRC patients. As a result, there is a pressing need for the development of new therapies to address metastatic disease.

Cetuximab, a monoclonal antibody that targets the EGFR, is a mainstream treatment in immunotherapy for mCRC. Despite its established efficacy in improving survival outcomes, its high cost, along with potential severe adverse effects such as skin toxicity and gastrointestinal disorders, has led to concerns among patients and healthcare providers (Rimassa et al., 2019; Kasper et al., 2020). To address some of these challenges, Cetuximab-β—a modified version of the original drug—was developed, aiming to offer a more cost-effective and tolerable alternative (FOLFIRI, 2024) (Shi et al., 2019). However, there remains a significant gap in the study, with no direct comparisons of the cost-effectiveness between Cetuximab-β and Cetuximab. This study aims to fill this gap by evaluating the economic impact of Cetuximab-β in combination with FOLFIRI, comparing it with the combination of Cetuximab. The findings of this analysis will provide valuable insights for healthcare policy and resource allocation decisions for RAS/BRAF WT mCRC patients in China, supporting evidence-based decision-making for policymakers.In the NMA, no significant differences in PFS or OS were found between Cetuximab-β and Cetuximab, suggesting equivalent efficacy. In terms of safety, Cetuximab-β was associated with a lower incidence of hypokalemia and rash compared to Cetuximab, although these differences were not statistically significant. However, diarrhea occurred significantly less frequently with Cetuximab-β. Hematologic toxicities, including neutropenia and febrile neutropenia, were higher common with Cetuximab-β, but this difference was not statistically significant. Since hematologic toxicities were primarily chemotherapy-related, we conclude that Cetuximab-β offers comparable efficacy and a superior safety profile compared to Cetuximab.

In the cost-effectiveness analysis, Cetuximab-β improved effectiveness by 0.10 QALYs and reduced overall costs by $12,005.54 compared to Cetuximab, resulting in an ICER of -$120,742.96 per QALY. Although the NMA found no statistically significant difference in OS (HR for Cetuximab vs. Cetuximab-β = 1.10, 95% CI 0.67–1.90), the PSM extrapolation projected a numerically longer mean OS duration for Cetuximab-β (2.73 LYs vs. 2.54 LYs). Over the 10-year time horizon, this small difference cumulatively resulted in a gain in LYs, which translated to the incremental 0.10 QALY gain. The cost reduction associated with Cetuximab-β primarily arises from lower drug costs. Additionally, Cetuximab-β not only extends LYs for patients with mCRC but also reduces the cost of managing AEs compared to Cetuximab. These findings suggest that Cetuximab-β is more effective and cost-effective than Cetuximab, with an ICER that falls within the cost-effective range. Sensitivity analyses revealed that the cost difference between Cetuximab-β and Cetuximab was a key determinant of cost-effectiveness, with price sensitivity being the most influential factor. This was further confirmed in a separate study assuming identical efficacy and safety, which demonstrated that mCRC patients treated with Cetuximab-β plus FOLFIRI saved $10,567.38 compared to those receiving Cetuximab plus chemotherapy.

Our findings challenge the presumption that novel biologics inevitably increase healthcare expenditures by demonstrating Cetuximab-β′s unique cost-saving profile, while reconciling divergent conclusions from prior Chinese cost-effectiveness analyses. Notabely, Cetuximab-β fundamentally differs from originator Cetuximab in its economic performance: (Wang et al., 2020) reported unfavorable economics for originator Cetuximab + FOLFOX (ICER $164,044/QALY), a divergence attributable not only to chemotherapy backbone differences (FOLFOX’s higher cost vs. FOLFIRI), but more critically to Cetuximab-β′s intrinsic price advantage. Crucially, while Wu et al. (2017) showed originator Cetuximab’s cost-effectiveness required patient assistance programs (PAP; ICER $14,049/QALY), Cetuximab-β achieves dominance without subsidies through its biosimilar pricing—resolving the accessibility barrier that plagued originator biologics in resource-limited settings. As the first Asian CEA of this specific biosimilar, our work positions cetuximab-β as a self-sustaining solution to China’s “efficacy-cost paradox.”

Our findings indicate that at China’s WTP threshold ($37,023/QALY), Cetuximab-β is highly likely to be cost-effective, with direct per-patient savings of $12,005 (equivalent to 32.4% of China’s GDP *per capita*). This robust economic advantage presents a significant opportunity for inclusion in national reimbursement lists. Unlike PAP-dependent originator biologics, cetuximab-β′s inherent affordability establishes a sustainable biosimilar pricing paradigm that aligns with DRG payment reforms—enabling hospitals to maintain therapeutic efficacy while reducing episode-based costs. Resources liberated by Cetuximab-β adoption could be strategically reallocated to address systemic gaps in CRC screening, directly combating the late-stage diagnoses that drive metastatic burden.

### Limitations

This study has several limitations. First, due to the lack of direct head-to-head comparison studies between Cetuximab-β and Cetuximab, we conducted a NMA to indirectly assess their efficacy and safety. However, clinical and methodological differences across the included studies, such as variations in patient populations and study designs, may introduce bias (Seitidis et al., 2022; Yun et al., 2023). We compared baseline characteristics across studies and found few clinically significant differences, which suggests that our results are relatively stable. Additionally, the use of a random-effects model helps mitigate potential bias. Second, while Cetuximab can be combined with either FOLFIRI or FOLFOX, Cetuximab-β is typically combined only with FOLFIRI. Consequently, our analysis compares Cetuximab-β with FOLFIRI and Cetuximab with either FOLFIRI or FOLFOX, which may introduce bias. However, previous studies have reported similar efficacy and safety profiles for FOLFIRI and FOLFOX (Yoshino et al., 2024; Zhou et al., 2023), suggesting that this bias is unlikely to significantly affect the results. Third, we inferred the long-term survival benefit based on short-term survival data from the included studies. However, these estimates may change with longer follow-up, representing an inherent limitation of our model. Thus, it is necessary to validate these health outcomes using real-world data to assess the model’s accuracy.

## Conclusion

In brief, Cetuximab-β plus FOLFIRI demonstrated equivalent efficacy in prolonging OS and PFS compared to Cetuximab plus chemotherapy, but it exhibited a better safety profile. Additionally, Cetuximab-β plus FOLFIRI was found to be a more cost-effective first-line treatment strategy for mCRC in China, with a WTP threshold of $36,994 per QALY, compared to Cetuximab plus chemotherapy. These findings can guide clinicians in selecting optimal treatment strategies and inform reimbursement policies.

## Data Availability

The original contributions presented in the study are included in the article/Supplementary Material, further inquiries can be directed to the corresponding authors.
