# Supplementary material for: Network meta-analysis and cost-effectiveness analysis comparing Cetuximab-β and Cetuximab for Chinese patients with RAS/BRAF wild-type metastatic colorectal cancer
Source: Front Pharmacol. 2025 Sep 15;16:1568385. doi: 10.3389/fphar.2025.1568385 (PMC12477409; doi:10.3389/fphar.2025.1568385)
Supplement: Supplementary file 1 [file DataSheet1.docx]

**Table S1. Treatment options and cost during PD.**

| **Treatments** | **Cost per cycle**, $ | **Proportion of clinical use** |
| --- | --- | --- |
| Bevacizumab + FOLFIRI | 1583.79 | 27.78% |
| Bevacizumab + FOLFOX | 1452.41 | 11.57% |
| Bevacizumab + Fluorouracil | 1188.39 | 4.17% |
| Fluorouracil | 239.15 | 6.02% |
| FOLFOX | 634.55 | 24.54% |
| No Treatments | 0.00 | 25.93% |

PD, progressive disease; FOLFIRI, calcium folinate + fluorouracil + irinotecan; FOLFOX, calcium folinate + fluorouracil + oxaliplatin.

The date of Proportion of clinical use came from Han 2020*.*^[^^[[1]](#endnote-1)]^

**Table S2. Parameters of OS distribution and AIC and BIC values in Cetuximab-β plus FOLFIRI group.**

| **Models** | **Parameters** | | | **AIC** | **BIC** |
| --- | --- | --- | --- | --- | --- |
| **Exponential** | **rate=lambda** |  |  | 976.86 | 980.409 |
|  | 0.023 |  |  |  |  |
| **WeibullPH** | **shape=a** | **scale=lambda** |  | **967.765** | **974.863** |
|  | 1.342 | 0.008 |  |  |  |
| **Gompertz** | **shape=a** | **rate=b** |  | 967.903 | 975.001 |
|  | 0.032 | 0.015 |  |  |  |
| **Log-normal** | **meanlog=mu** | **sdlog=sigma** |  | 981.947 | 989.045 |
|  | 3.409 | 1.251 |  |  |  |
| **Log-logistic** | **shape=a** | **scale=b** |  | 972.025 | 979.123 |
|  | 1.552 | 28.509 |  |  |  |
| **Generalized Gamma** | **mu** | **sigma** | **Q** | 969.045 | 979.692 |
|  | 3.672 | 0.586 | 1.449 |  |  |

OS, overall survival; AIC, Akaike's information criterion; BIC, Bayesian information criterion.

This table evaluates six statistical models for OS distribution in the Cetuximab-β plus FOLFIRI group, using AIC and BIC to compare goodness-of-fit. The Weibull proportional hazards (WeibullPH) model demonstrated the lowest AIC (967.765) and BIC (974.863), indicating the best fit among the models analyzed. In contrast, the Log-normal model had the highest AIC (981.947) and BIC (989.045), reflecting a poorer fit. Based on these results, the WeibullPH model was selected for the OS due to its superior performance in describing the data.

**Table S3. Parameters of PFS distribution and AIC and BIC values in Cetuximab-β plus FOLFIRI group.**

| **Models** | **Parameters** | | | **AIC** | **BIC** |
| --- | --- | --- | --- | --- | --- |
| **Exponential** | **rate=lambda** |  |  | 976.86 | 980.409 |
|  | 0.052 |  |  |  |  |
| **WeibullPH** | **shape=a** | **scale=lambda** |  | **967.765** | **974.863** |
|  | 1.788 | 0.008 |  |  |  |
| **Gompertz** | **shape=a** | **rate=b** |  | 967.903 | 975.001 |
|  | 0.091 | 0.026 |  |  |  |
| **Log-normal** | **meanlog=mu** | **sdlog=sigma** |  | 981.947 | 989.045 |
|  | 2.492 | 0.836 |  |  |  |
| **Log-logistic** | **shape=a** | **scale=b** |  | 972.025 | 979.123 |
|  | 2.233 | 12.182 |  |  |  |
| **Generalized Gamma** | **mu** | **sigma** | **Q** | 969.045 | 979.692 |
|  | 2.732 | 0.557 | 1.009 |  |  |

PFS, progression-free survival; AIC, Akaike's information criterion; BIC, Bayesian information criterion.

This table compares six statistical models for PFS distribution in the Cetuximab-β plus FOLFIRI group using AIC and BIC, both of which evaluate model fit by balancing goodness of fit and complexity. The WeibullPH model demonstrated the lowest AIC (967.765) and BIC (974.863), indicating the best fit among the models assessed. In contrast, the Log-normal model had the highest AIC (981.947) and BIC (989.045), reflecting a poorer fit. Consequently, the WeibullPH model was selected for its optimal performance in describing the PFS distribution.

**Table S4. Cost of drug administration.**

| **Items** | **Treatments** | **Cost per per cycle, $** |
| --- | --- | --- |
| Venous transfusion | Cetuximab-β, Cetuximab | 53.28 |
|  | FOLFIRI, FOLFOX | 79.92 |
| Drug configuration | Cetuximab-β, Cetuximab | 120.60 |
|  | FOLFIRI, FOLFOX | 42.00 |
| Pump assisted intravenous infusion | Cetuximab-β, Cetuximab | 4.40 |
|  | FOLFIRI, FOLFOX | 6.60 |
| Sodium chloride injection | ALL | 21.84 |

The prices were derived from the average prices of medical service items of medical institutions in five cities in China (Beijing^[2]^, Guangzhou^[3]^, Wuhan^[4]^, Zhenjiang^[5]^, and Chengdu^[6]^).

**Table S5. Cost of follow-up and monitoring.**

| **Items** | **Consumption per cycle** | **Cost per cycle, $** |
| --- | --- | --- |
| Outpatient consultation | 1 | 2.57 |
| Complete blood count | 1 | 2.15 |
| Liver and renal function tests | 1 | 8.58 |
| Routine urine test | 1 | 1.68 |
| Electrolyte test | 1 | 1.79 |
| Chest, abdominal, and pelvic CT scans | 1 | 83.90 |
| Tumor marker test | 1 | 9.61 |
| Electrocardiograph | 1 | 2.33 |

The prices were derived from the average prices of medical service items of medical institutions in five cities in China (Beijing^[^^[[2]](#endnote-2)]^, Guangzhou^[^^[[3]](#endnote-3)]^, Wuhan^[^^[[4]](#endnote-4)]^, Zhenjiang^[^^[[5]](#endnote-5)]^, and Chengdu^[^^[[6]](#endnote-6)]^).

**Table S6. Basic characteristics of included studies in Network Meta-Analysis.**

| First author | Publication year | Type of trial | Region | Treatment group | Control group | No. of patients | Outcome Measures |
| --- | --- | --- | --- | --- | --- | --- | --- |
| Shi^[[7]](#endnote-7)^ | 2022 | III | China | Cetuximab-β+FOLFIRI | FOLFIRI | 505 | ①②③ |
| Qin^[[8]](#endnote-8)^ | 2018 | III | China | Cetuximab+FOLFOX | FOLFOX | 393 | ①②③ |
| Bokemeyer^[[9]](#endnote-9)^ | 2009 | II | Europe | Cetuximab+FOLFOX | FOLFOX | 134 | ①③ |
| Tveit^[[10]](#endnote-10)^ | 2012 | III | Northern Europe | Cetuximab+FOLFOX | FOLFOX | 194 | ②③ |
| Bokemeyer^[[11]](#endnote-11)^ | 2011 | II | Germany | Cetuximab+FOLFOX | FOLFOX | 164 | ①②③ |
| Van^[[12]](#endnote-12)^ | 2015 | II | Globally | Cetuximab+FOLFIRI | FOLFIRI | 315 | ①②③ |

① PFS; ② OS; ③ Grade 3/4 adverse events (AEs).

Six clinical trials were included in the network meta-analysis (NMA). Among these, four were phase III trials, and two were phase II trials. The treatment groups included cetuximab-β plus FOLFIRI and cetuximab combined with either FOLFIRI or FOLFOX, while the control groups consisted of FOLFIRI in two trials and FOLFOX in four trials. Sample sizes ranged from 134 to 505 patients, with a median of approximately 259, reflecting variability across the studies. Key outcome indicators included PFS (assessed in five trials), OS (assessed in four trials), and grade 3/4 AEs (assessed in five trials), providing robust data for the comparative evaluation of these regimens.

**Table S7. The baseline characteristics of the NMA included studies.**

| **Source** | **Shi 2022^2^** | | **Qin 2018^3^** | | **Bokemeyer 2009^4^** | | **Tveit 2012^5^** | | **Bokemeyer 2011^6^** | | **Van 2015^7^** | |
| --- | --- | --- | --- | --- | --- | --- | --- | --- | --- | --- | --- | --- |
| Group | Treatment group | Control group | Treatment group | Control group | Treatment group | Control group | Treatment group | Control group | Treatment group | Control group | Treatment group | Control group |
| No. of patients | (n=257) | (n=248) | (n=193) | (n=200) | (n=61) | (n=73) | (n=97) | (n=97) | (n=72) | (n=92) | (n=178) | (n=189) |
|  | No. | % | No. | % | No. | % | No. | % | No. | % | No. | % |
| Gender | | | | | | | | | | | | |
| Male | 179 | 70 | 170 | 69 | 127 | 66 | 139 | 70 | 30 | 49 | 44 | 60 |
| Female | 78 | 30 | 78 | 31 | 66 | 34 | 61 | 31 | 31 | 51 | 29 | 40 |
| Age, years | | | | | | | | | | | | |
| Median | 57 | 58 | 56 | 56 | 59 | 59 | 60 | 60 | 59 | 63 | 60 | 59 |
| Range | 23-74 | 23-75 | 21-83 | 21-78 | 24-74 | 36-82 | 35-75 | 24-74 | 24-75 | 37-82 | 24-79 | 19-82 |
| ECOG performance status | | | | | | | | | | | | |
| 0 | 85 | 33 | 81 | 33 | 63 | 33 | 66 | 33 | 19 | 31 | 27 | 37 |
| 1 | 172 | 67 | 167 | 67 | 130 | 67 | 134 | 67 | 37 | 61 | 37 | 51 |
| 2 | - | - | - | - | - | - | - | - | 5 | 8 | 9 | 12 |
| Liver metastasis only | 179 | 70 | 189 | 76 | 193 | 100 | 200 | 100 | 54 | 89 | 65 | 89 |
| Organs with metastases | | | | | | | | | | | | |
| ≤ 2 | 180 | 70 | 154 | 62 | 153 | 79 | 143 | 72 | 49 | 80 | 55 | 75 |
| > 2 | 77 | 30 | 94 | 38 | 40 | 21 | 57 | 29 | 12 | 20 | 18 | 25 |

This table summarizes the baseline characteristics of patients included in the NMA. Gender and age showed relatively low variability, with male representation ranging from 49% to 70% and median age spanning 56 to 63 years across studies. In contrast, substantial heterogeneity was observed in ECOG performance status (ECOG 0: 19%–73%, ECOG 1: 22%–67%) and the proportion of patients with liver-only metastases (54%–100%). Variability was also noted in the number of metastatic organs, with patients having ≤2 metastatic organs being more common (55%–88%), while the proportion with >2 organs ranged from 10% to 57%. To address these differences and account for study-level variability, a random-effects model was applied in subsequent analyses, ensuring more robust and reliable results.

**Figure S1. Structure of the partitioned survival model.**


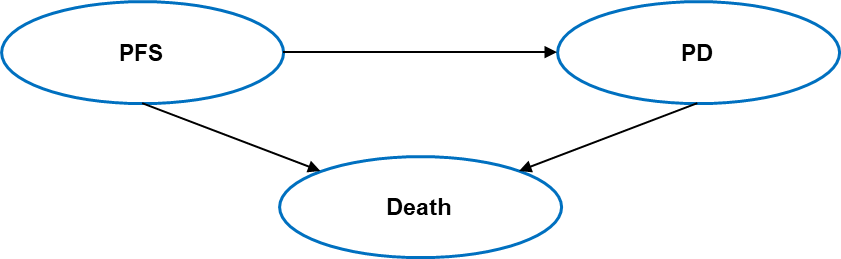


PFS, progression-free survival; PD, progressive disease.

The model consisted of three health states: PFS, PD, and Death. A partitioned survival model was employed to simulate OS and PFS for different treatment regimens. Survival rates at each cycle were derived from the OS curve, while the number of patients in the PFS state was determined using the PFS curve. Patients in the PD state were calculated as the difference between the total number of survivors and those in the PFS state, and the Death state included all remaining patients who had not survived. Patients enter the model in the PFS state and transition sequentially through the states of PFS, PD, and Death over time. The model terminates when the majority of patients reach the Death state, and final cost and outcome estimates are based on the distribution of patients across the health states.

**Figure S2. Model Fitting Analysis of OS.**


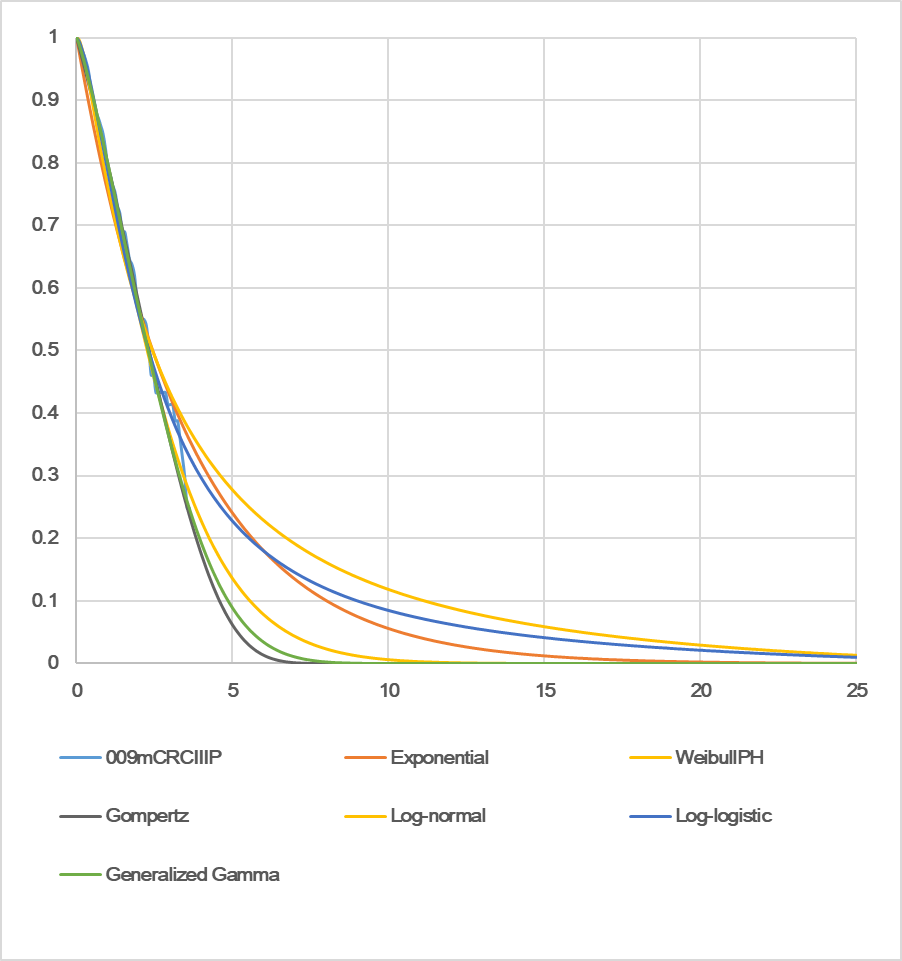


**Figure S3. Model Fitting Analysis of PFS.**


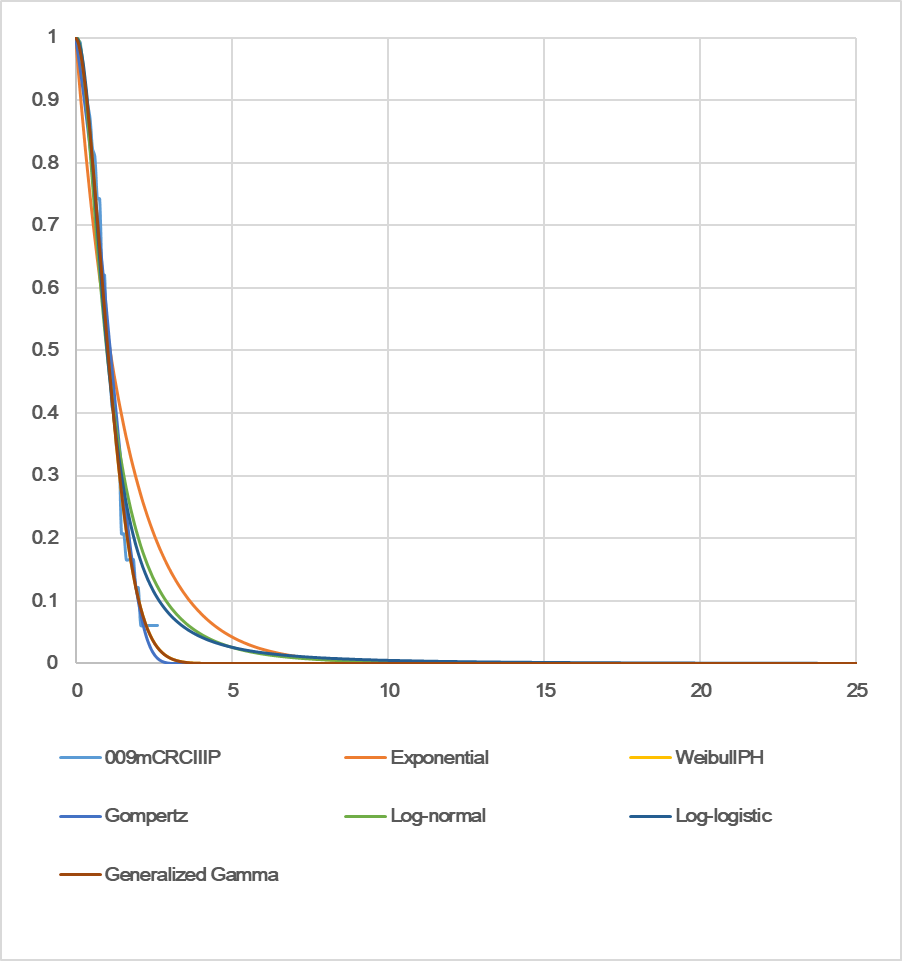


**Figure S4. Survival Analysis of OS.**


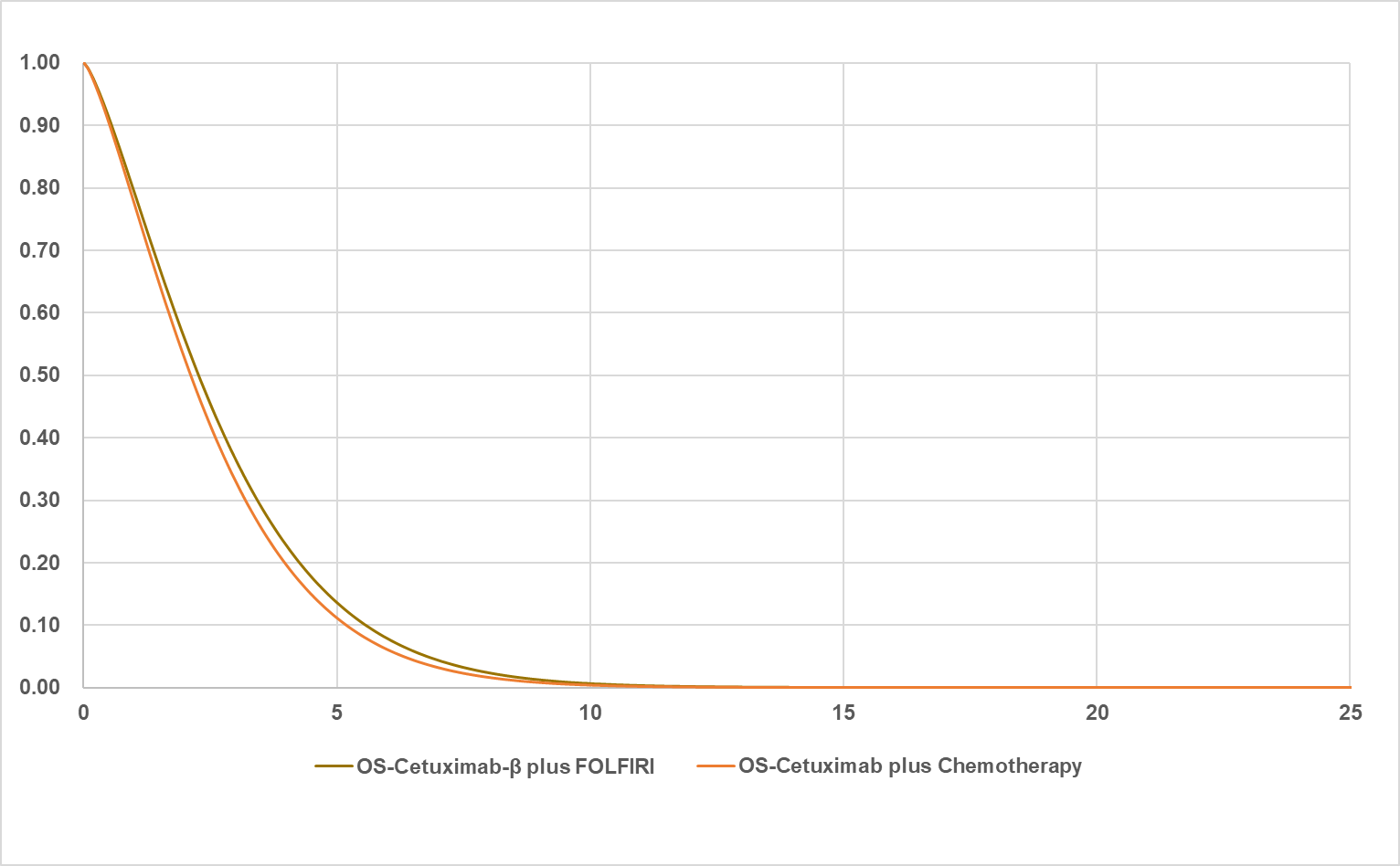


**Figure S5. Survival Analysis of PFS.**


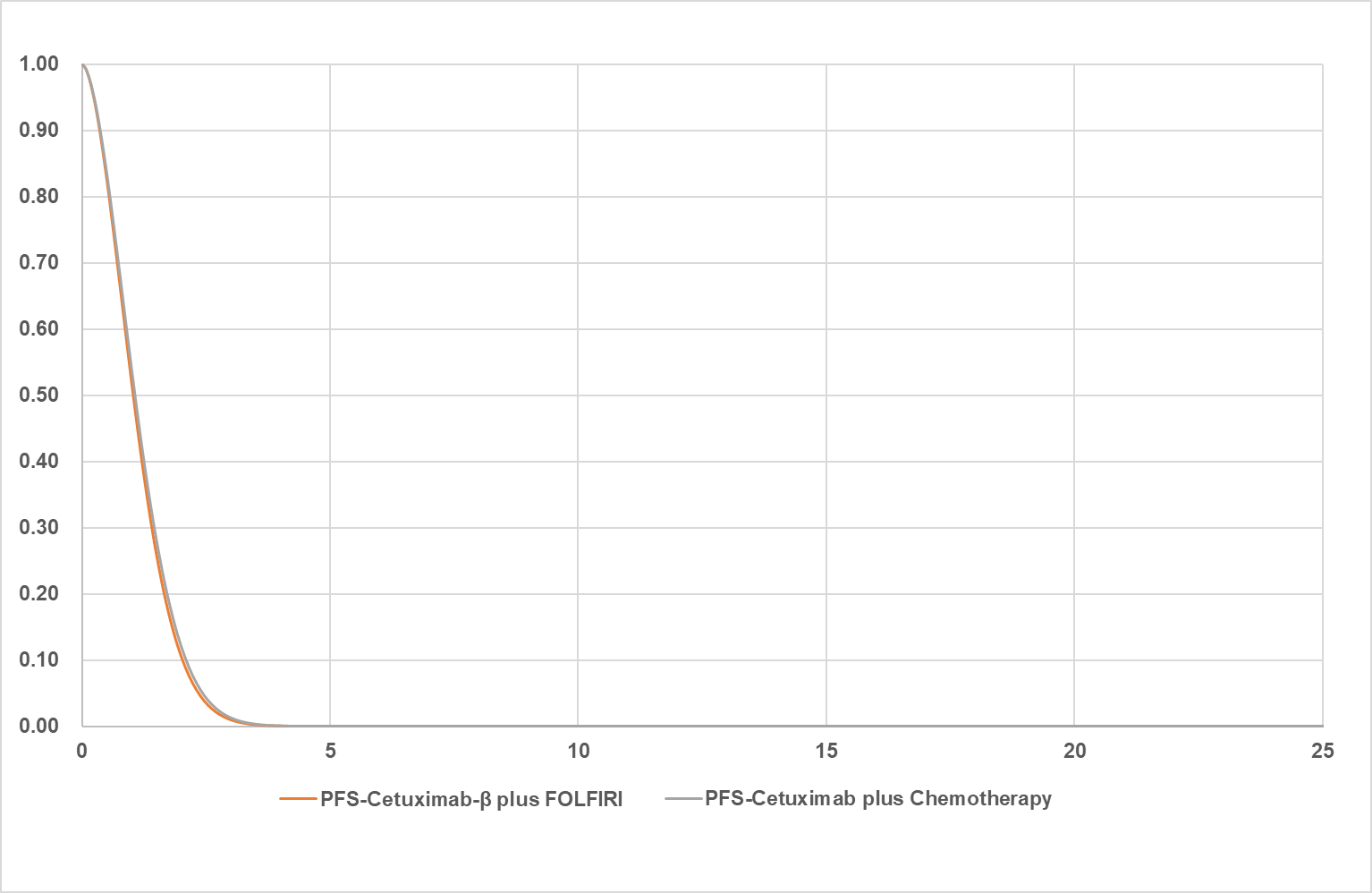


**Figure S6. Flowchart of Study Selection.**


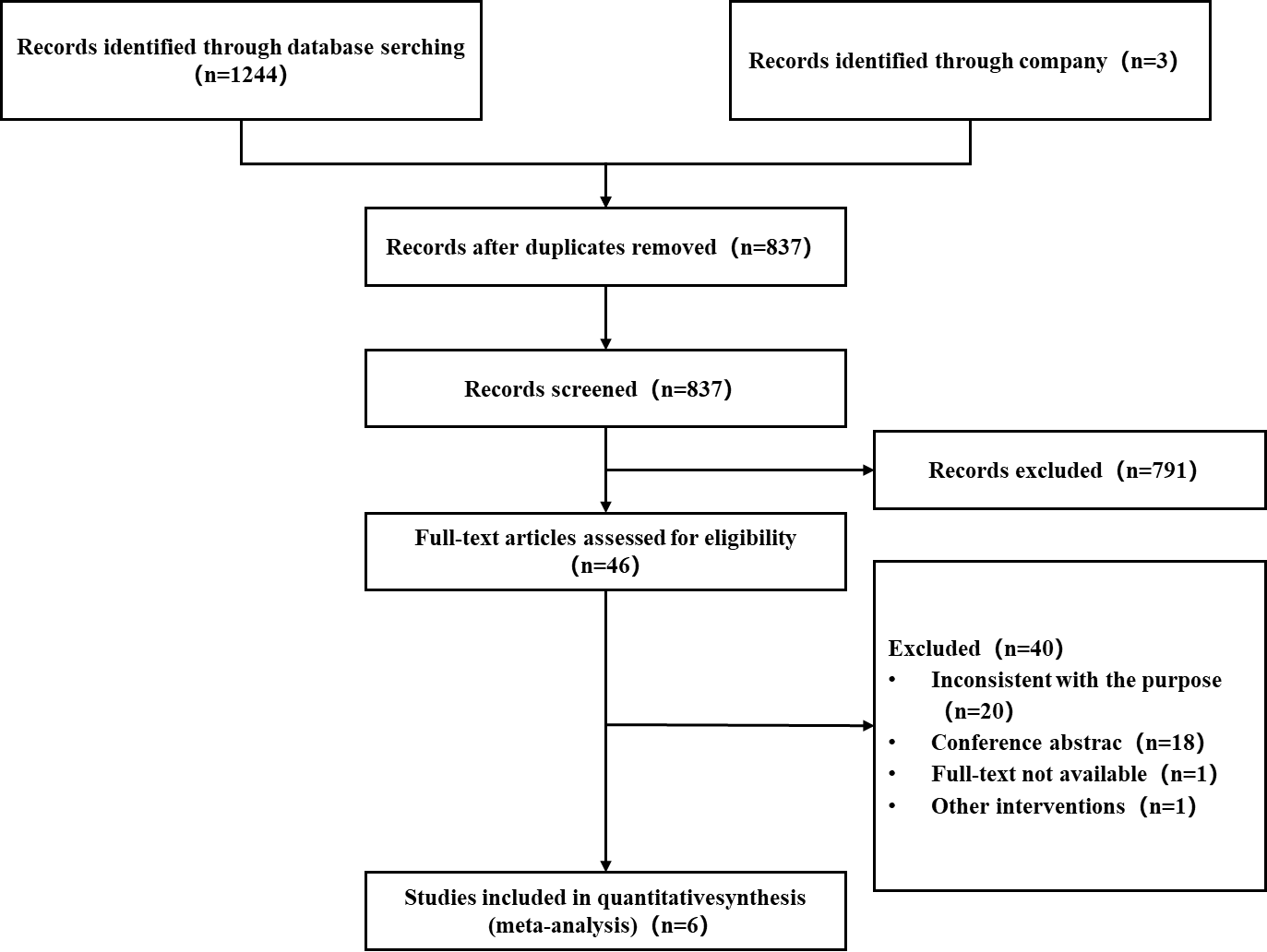


**Figure S7. Model Schematic for** **Network Meta-analysis**


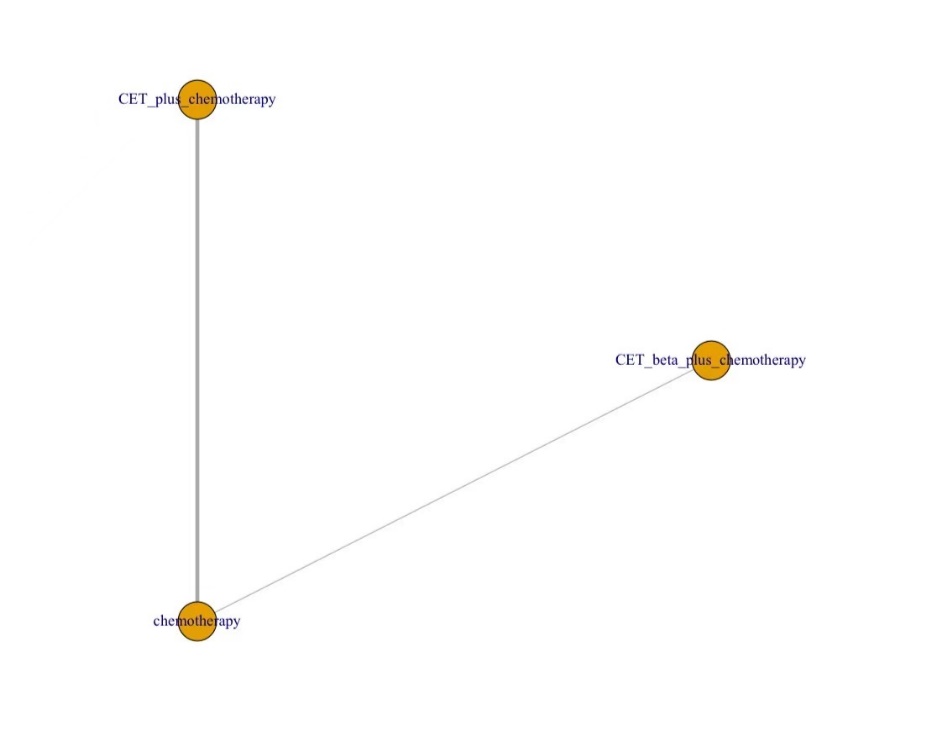


CET, Cetuximab; CET_beta, Cetuximab-β.

**Figure S8. Risk of Bias Summary.**


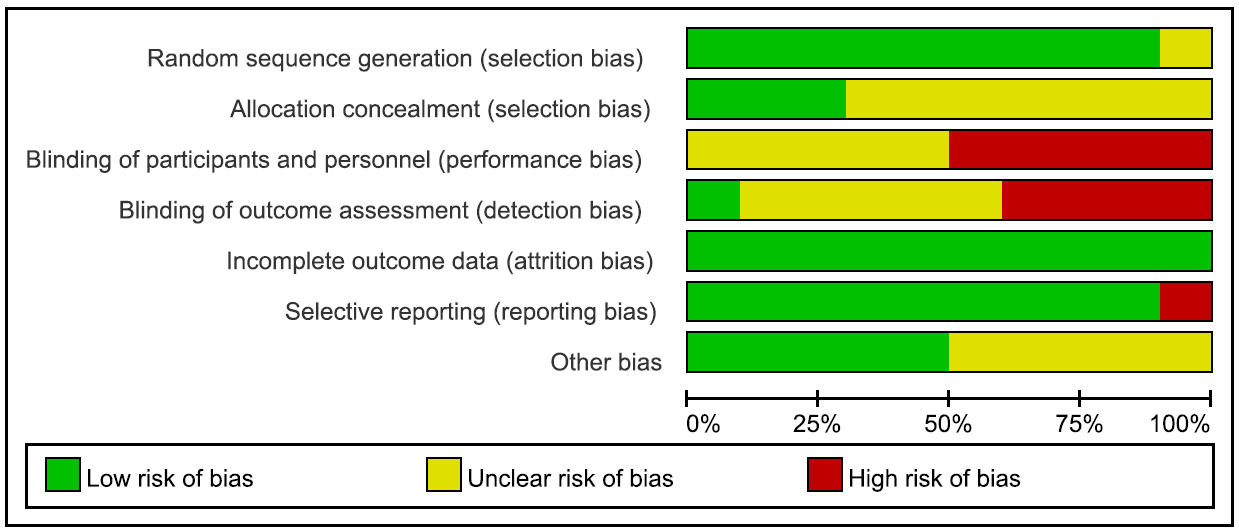


The six studies included in the Network Meta-analysis were generally of high quality, with most classified as 'low risk' or 'uncertain' for the seven major sources of bias, including incomplete outcome data, which had the highest proportion of 'low risk' ratings. In contrast, blinding of patients and trial personnel was most frequently classified as 'high risk.' Given that the combination of Cetuximab with chemotherapy is clearly superior to chemotherapy alone, implementing double-blind controls would be ethically questionable; thus, blinding was not used in any of the studies.

**Figure S9. Scenario analyses results of time horizon from 10 to 35 years.**


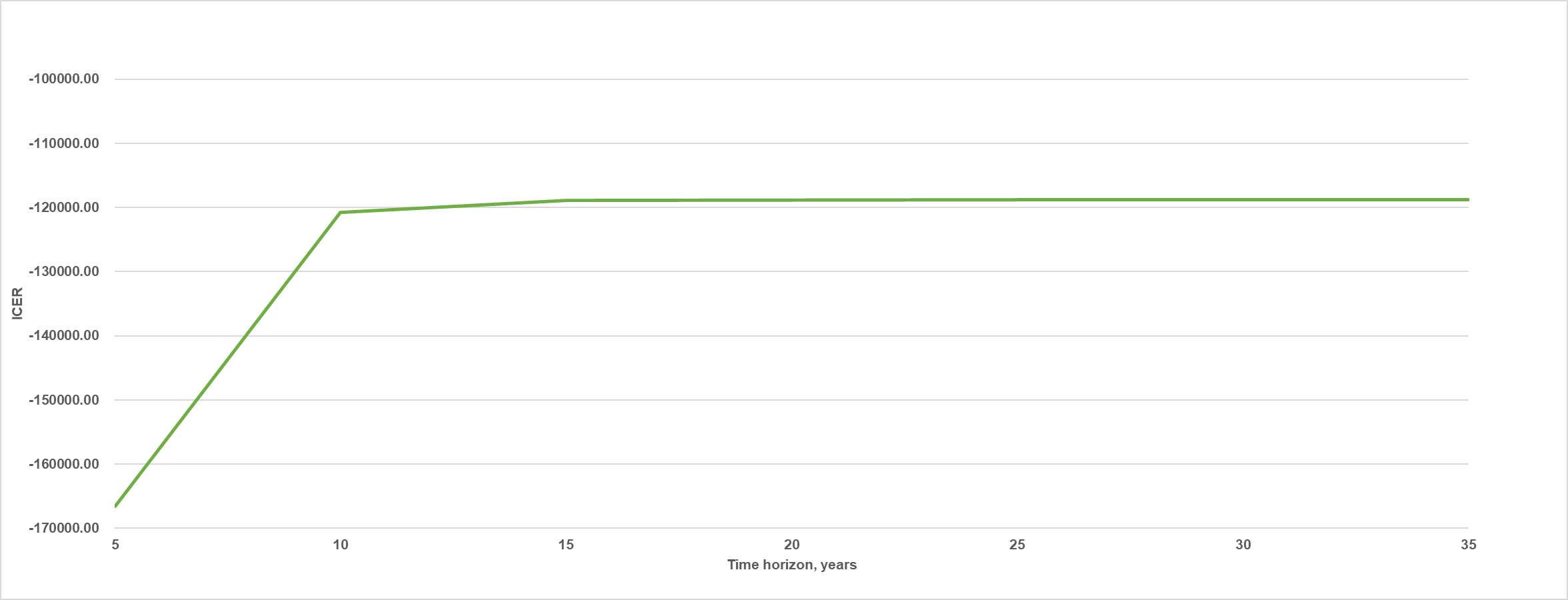


At shorter time horizons, the ICER (Incremental Cost-Effectiveness Ratio) exhibits considerable variability, with the steepest decline observed at the 5-year mark. By the 10-year horizon, the ICER stabilizes, with negligible changes when extended up to 35 years. This stabilization suggests that the incremental costs and benefits of the treatment are sufficiently captured within a 10-year period, making it the appropriate base case for analysis. A 10-year horizon effectively balances the need for comprehensive long-term outcome assessment with practical modeling constraints, consistent with standard practices in pharmacoeconomic evaluations.

**References**

1. [] Han, J., Xiao, D., Tan, C., et al. (2020). Cost-Effectiveness Analysis of First-Line FOLFIRI Combined With Cetuximab or Bevacizumab in Patients With RAS Wild-Type Left-Sided Metastatic Colorectal Cancer. Cancer control : journal of the Moffitt Cancer Center, 27(1), 1073274820902271. [↑](#endnote-ref-1)
2. [] BeiJin Medical Security Bureau. Medical Service Price Inquiry. Accessed July 2024. https://ybj.beijing.gov.cn/2020_zwfw/2020_bmcx/202002/P020211231647015053723.xls [↑](#endnote-ref-2)
3. [] Guangzhou Medical Security Bureau. Summary Table of Basic Medical Service Item Prices for Public Medical Institutions in Guangzhou. Accessed July 2024. https://www.gz.gov.cn/gzybj/gkmlpt/content/10/10055/post_10055305.html#14609 [↑](#endnote-ref-3)
4. [] Wuhan Medical Security Bureau. Medical Service Item Prices of Public Medical Institutions in Wuhan. Accessed July 2024. https://ybj.wuhan.gov.cn/bsfw/yyfwjgyzbcg/202407/t20240718_2430221.shtml. [↑](#endnote-ref-4)
5. [] Zhenjiang Medical Security Bureau. Medical Service Price Inquiry. Accessed July 2024. https://ybj.jiangsu.gov.cn/art/2022/7/6/art_85491_10531639.html. [↑](#endnote-ref-5)
6. [] Chengdu Medical Security Bureau. Medical Service Price Inquiry. Accessed July 2024. https://cdyb.chengdu.gov.cn/ylbzj/c149273/new_list.shtml. [↑](#endnote-ref-6)
7. [] CMAB009 combined with FOLFIRI first-line treatment in patients with RAS/BRAF wild-type, metastatic colorectal cancer. ClinicalTrials.gov. Updated April 10, 2024. Accessed June 26, 2024. https://clinicaltrials.gov/study/NCT03206151. [↑](#endnote-ref-7)
8. [] Qin, S., Li, J., Wang, L., et al. (2018). Efficacy and Tolerability of First-Line Cetuximab Plus Leucovorin, Fluorouracil, and Oxaliplatin (FOLFOX-4) Versus FOLFOX-4 in Patients With RAS Wild-Type Metastatic Colorectal Cancer: The Open-Label, Randomized, Phase III TAILOR Trial. Journal of clinical oncology : official journal of the American Society of Clinical Oncology, 36(30), 3031–3039. [↑](#endnote-ref-8)
9. [] Bokemeyer, C., Bondarenko, I., Makhson, A., et al. (2009). Fluorouracil, leucovorin, and oxaliplatin with and without cetuximab in the first-line treatment of metastatic colorectal cancer. Journal of clinical oncology : official journal of the American Society of Clinical Oncology, 27(5), 663–671. [↑](#endnote-ref-9)
10. [] Tveit, K. M., Guren, T., Glimelius, B., et al. (2012). Phase III trial of cetuximab with continuous or intermittent fluorouracil, leucovorin, and oxaliplatin (Nordic FLOX) versus FLOX alone in first-line treatment of metastatic colorectal cancer: the NORDIC-VII study. Journal of clinical oncology : official journal of the American Society of Clinical Oncology, 30(15), 1755–1762. [↑](#endnote-ref-10)
11. [] Bokemeyer, C., Bondarenko, I., Hartmann, J. T., et al. (2011). Efficacy according to biomarker status of cetuximab plus FOLFOX-4 as first-line treatment for metastatic colorectal cancer: the OPUS study. Annals of oncology : official journal of the European Society for Medical Oncology, 22(7), 1535–1546. [↑](#endnote-ref-11)
12. [] Van Cutsem, E., Lenz, H. J., Köhne, C. H., et al. (2015). Fluorouracil, leucovorin, and irinotecan plus cetuximab treatment and RAS mutations in colorectal cancer. Journal of clinical oncology : official journal of the American Society of Clinical Oncology, 33(7), 692–700. [↑](#endnote-ref-12)
